# The contribution of home-based technology to older people's quality of life in extra care housing

**DOI:** 10.1186/1471-2318-11-68

**Published:** 2011-10-31

**Authors:** Hossein Matlabi, Stuart G Parker, Kevin McKee

**Affiliations:** 1 The Medical Education Research Centre, R & D Campus, Tabriz University of Medical Sciences, Daneshgah Ave., Tabriz, P.C.: 5165665811, Iran; 2 Department of Health Education and Promotion, Faculty of Health and Nutrition, Tabriz University of Medical Sciences, Tabriz, P.C.: 5166614711, Iran; 3 Sheffield Institute for Studies on Ageing, The University of Sheffield, 217 Portobello, Sheffield S1 4DP, UK; 4 School of Health and Social Studies, Dalarna University, 791 88 Falun, Sverige, Sweden; 5 Gerontology Centre, Dalarna Research Institute, Myntgatan 2, 79151 Falun, Sweden

**Keywords:** Home-Based Technology, Older People, Assistive Technology, Quality of Life, Well-Being, Extra Care Housing

## Abstract

**Background:**

British government policy for older people focuses on a vision of active ageing and independent living. In the face of diminishing personal capacities, the use of appropriate home-based technology (HBT) devices could potentially meet a wide range of needs and consequently improve many aspects of older people's quality of life such as physical health, psychosocial well-being, social relationships, and their physical or living environment. This study aimed to examine the use of HBT devices and the correlation between use of such devices and quality of life among older people living in extra-care housing (ECH).

**Methods:**

A structured questionnaire was administered for this study. Using purposive sampling 160 older people living in extra-care housing schemes were selected from 23 schemes in England. A face-to-face interview was conducted in each participant's living unit. In order to measure quality of life, the SEIQoL-Adapted and CASP-19 were used.

**Results:**

Although most basic appliances and emergency call systems were used in the living units, communally provided facilities such as personal computers, washing machines, and assisted bathing equipment in the schemes were not well utilised. Multiple regression analysis adjusted for confounders including age, sex, marital status, living arrangement and mobility use indicated a coefficient of 1.17 with 95% CI (0.05, 2.29) and *p *= 0.04 [SEIQoL-Adapted] and 2.83 with 95% CI (1.17, 4.50) and *p *= 0.001 [CASP-19].

**Conclusions:**

The findings of the present study will be value to those who are developing new form of specialised housing for older people with functional limitations and, in particular, guiding investments in technological aids. The results of the present study also indicate that the home is an essential site for developing residential technologies.

## Background

It has been estimated that older people spend 80-90 per cent of their time in their home. Although housing type and condition are widely considered to be important contributors to health and quality of life, many older people still live in unsuitable housing [1].

Furthermore, 19 per cent of men and 33 per cent of women aged 65-74 lived alone in the UK in 2005, whilst 29 per cent of men and 60 per cent of women aged 75 and over lived alone [2]. The World Health Organisation [3] and the Royal College of General Practitioners [4] described older people living alone as an at-risk group, which should be targeted for specific attention. They require more home visits, make more use of community services [5] and are most likely to be depressed, lonely, and unhappy [6].

The use of technology to support independent living is mentioned explicitly in many recent government documents. Policy for older people in the UK and in EU countries, particularly in Spain and the Scandinavian countries, and in Australia, focuses on a vision of active ageing and independent living by providing modern and 'person-centred' services to meet their needs, helping them to live in the community as long as possible, and by supporting their carers. One component of an effective policy on active ageing and independent living is the provision of appropriate built environments that take the special needs of older people into consideration [7].

Additionally, in most developed countries there is increasing interest in and financial support for new models of housing. For example, the Department of Health for England provided investment through the Extra Care Housing (ECH) Funding Initiative of £87 million in 2004-06, £60 million in 2006-08 and £80 million in 2008-2010 to local authorities and their partners to construct new living units for older people [8].

ECH has no single universally accepted definition, and indeed its use is presently restricted to the United Kingdom. An equivalent term in North America and Australia is 'housing with care' or 'assisted living' (AL). ECH is specially designed housing with more personal care, more communal space and facilities than are found in a traditional sheltered housing scheme but without the institutional features of a care home. Setting decent standards of housing and services within ECH would enable older people to remain in their homes for much longer than would be the case in more conventional forms of sheltered housing [9].

As older people are heavy users of health and social services, residential technology may reduce unnecessary hospital admissions, support hospital discharge, and provide intermediate care [10]. The use of appropriate devices could potentially meet a wide range of needs and consequently improve many aspects of older people's quality of life such as physical health, psychosocial well-being, social relationships, and their physical or living environment [11].

### Home-based technology

In the future, new technologies and particularly domestic appliances will inevitably play an increasing role in many aspects of our lives. In its simplest and practical terms, technology can be defined as any device or system that controls and manages the physical environment [12]. In practice, as Agree and colleagues [[13], p.270] believed, *"the distinction between assistive technology, other household technology, and the environmental modifications may not always be clear"*. Assistive Technology (AT) is *"an umbrella term for any device or system that allows an individual to perform a task" *[[14], p.325]. AT can also be defined as *"any equipment or system that is used to maintain and improve the functional capabilities and independence of people with cognitive, physical or communication difficulties *[[15], *p.3]*. Furthermore, telecare facilities can be combined into a 'lifestyle reassurance package' with bed and chair occupancy sensors, passive infra-red movement detectors, a 'security package' that includes CCTV, intruder alarms, flood detectors, extreme heat detectors, a 'fall package' that comprises fall detectors, and finally, 'specialist devices' include wandering client systems, and epilepsy bed sensors [16].

HBT is here defined as technological devices that are owned or controlled by the household, such as kitchen appliances, personal computers, assistive technology, and telehealth monitors.

### The use of HBT devices and quality of life in older people

Various telecare, telemonitoring, telehealth and telemedicine technologies are now well established and widely used. They are used in combination with information and communication technologies (ICT) to deliver care and social services as with emergency alerts and remote monitoring [[7] and [10]]. Blackburn and colleagues [16] concluded that using household technology devices including 'lifestyle reassurance packages', 'fall packages', and 'specific devices' prevented older people going into hospital and could speed up hospital discharge by providing added support in their own home. The respondents in their study said that the devices gave peace of mind to their family members. They also experienced more social functioning and more satisfaction and security with pull-cord alarm systems and warden services.

The Scottish Executive developed telecare programmes for 75,000 people across Scotland, including 9,000 people with a diagnosis of dementia [17]. The study used equipment ranging from smoke alarms, flood and heat detectors, fall detectors and movement sensors to environmental controls. The findings revealed that the participants benefited from an increase in their independence at home. The residential technologies also postponed and diverted people from hospital and residential care admissions reduced.

The project Opening Doors for Older People [18] was launched to increase the level of care as needs increase, rather than moving the person into increasingly intensive care settings. Smart technologies, specifically a 'lifeline' unit, passive infrared detectors, flood detectors, heat sensors, and smoke detectors were installed in 1,950 newly built housing developments designed to offer housing with care with an onsite staff team for those who could not manage in their own homes. Nearly all the respondents in the study reported a positive impact of the smart technology, which had been important in relieving worries about falling and about home security.

The accumulated evidence indicates that HBT enhances older people's quality of life in several ways:

• Technology interventions help older people to remain in their comfortable setting and within a familiar community [19]. Moreover, staying at home would no longer involve social exclusion because of information and communication exchange through the Internet with the outside world [20].

• Assistive technology could help older people live independently and ease the challenges of caused by age or long-term chronic conditions by supporting daily living activities [20].

• Telecare potentially facilitates the delivery of far more customer focused housing, social and care services to people in their preferred environment [7].

• Assistive devices can reduce unnecessary hospital admissions, speed up hospital discharge, and provide intermediate care [10].

This paper reports a study of the use of HBT devices in ECH schemes and considers the connection between the use of such technology and the quality of life.

## Methods

### Hypothesis

There is an association between use of multiple HBT devices and quality of life among older people living in ECH schemes in England.

### Sampling and participants

Purposive sampling of ECH schemes was carried out to ensure that schemes of different size, type (new build, remodelled, private and public) and location were included in the study. Initially, 35 ECH schemes were chosen from the Elderly Accommodation Counsel's directory [21]. A power calculation, based on the SEIQoL index score as an outcome measure in a multiple regression model that assumed a medium effect size (*f*^2 ^= 0.15), a significance level of five per cent, and a power of 80 per cent, indicated a required sample of N = 109 scheme residents. However, given the clustered nature of the sample, and the need to ensure a broad representation of ECH schemes, it was decided to recruit 10 residents from each of 25 ECH schemes. Twelve schemes declined to take part in the study due to concerns about the over-testing of residents, and recruitment of participants across schemes varied due to local factors. Potential participants were ineligible if they had severe cognitive impairment or were too frail to undertake the survey. The achieved sample consisted of 160 older people, recruited from 23 ECH schemes located from across the breadth of England, a recruitment rate of 69.57%.

### Inclusion criteria for HBT items

In this study, HBT devices were categorised into two main types. First, *basic devices *include kitchen appliances (microwaves, electric kettles, toasters, electric hobs, washing machines), and lifts. Second, *assistive technology *such as personal computers (connected to the Internet), assisted-bathing facilities, electric window openers, emergency call systems, property exit sensors, automatic temperature thermostats, telehealth facilities and closed-circuit television (CCTV), by which images of the corridor outside a resident's front door are available to residents through their televisions.

The choice of devices was based on the ambition to take an integrated approach to the relationship between technologies and quality of life, on the checklist of the Department of Health for planning and equipping ECH [8] and on the results of the pilot study. Devices were selected that were particularly relevant to the five domains thought to influence adoption and use: health (telehealth facilities), safety (CCTV), social connectivity and legacy (personal computers) and contribution to others (alarms and detectors). Devices such as kettles, washing machines and lifts were also included to investigate the relationships between daily living activities and technology use. Moreover, the range of selected devices included equipment or technologies that are standard installations in ECH, such as assisted-bathing facilities, emergency call systems, and laundry/IT suites facilities.

It is important to note that lifts, assisted-bathing facilities and CCTV are communal facilities, while the others are appliances specific to individual living units. Some participants had their own personal computers and washing machines, and others had access to these devices in scheme computer rooms and launderettes respectively. Electric hobs and ovens were also standard equipment in the sampled ECH schemes.

### Materials/Reliability & validity

A quantitative-designed and structured questionnaire was administered for this study. Its feasibility, comprehensibility and reliability were tested among 21 residents living in five ECH schemes. Based on the results of the pilot study, the questionnaire was revised with a simplified SEIQoL scale and technical words, some aggregation of response options, naming additional devices, and reducing the number of items in scales. In addition, experience with the pilot led to the introduction of cue cards and some changes in the data recording.

The questionnaire was developed which incorporated CASP-19, the SEIQoL-Adapted, and the use of HBT devices. Other items contained in the questionnaire recorded information on participants' socio-demographic characteristics, including gender, age, marital status, ethnicity, overall health condition, living arrangement, mobility use, and scheme type, location and size.

CASP-19 is a subjective measure of well-being, which comprises of 19 Likert-type scaled items that cover four key life domains including control (C), autonomy (A), self-realisation (S), and pleasure (P). Each domain comprises four or five items in the form of statements that describe participants' potential feelings about their lives. For example, "*My age prevents me from doing the things I would like to do, I can do the things I want to do, I look forward to each day, and I feel full of energy these days*". Respondents can indicate how often a given statement is true for them: 'often', 'sometimes', 'not often', or 'never'. The range of overall score is between zero to 57, where a high score points towards good quality of life [22].

This scale was developed for a study of 286 British older people aged 65-75 years. Concerning internal consistency and validity, all domains had *"respectable internal homogeneity, good inter-domain correlations, and high loadings on a latent factor" *[[22], p.192]. Other previous studies of elderly people had confirmed that the CASP-19 has suitable validity for assessing their quality of life [22]. The CASP-19 was used by the English Longitudinal Study of Ageing and the British Household Panel Survey [23].

Schedule for the evaluation of individual quality of life-Direct Weighting (SEIQoL-DW) is based on social judgment theory that assesses quality of life from the individual's perspective. This measure derives from a structured interview that asks participants to nominate life domains that they consider as important to their own quality of life, weigh up their relative importance and their current level of satisfaction with each domain [24].

The SEIQoL is administrated in three stages. In the first stage, respondents are asked to think about their lives and nominate the five areas of life that are most important to their overall quality of life. Participants in the second stage rate each domain by its satisfaction score, with a range from worst possible (0) to best possible (100). The third stage is achieved by 'judgment analysis' for 30 randomly generated hypothetical scenarios to quantify the relative importance of each domain [25]. The total quality of life is then calculated by multiplying each domain weight (level of importance to the individual) by the individual's current self-rating on that particular domain (level of satisfaction) and summing these across the five domains [26].

As the SEIQoL instrument provides a feasible, valid and reliable assessment of quality of life, previous studies suggested that it could be used amongst elderly people, particularly those without severe physical difficulties or only mild cognitive impairment [27].

### SEIQoL-Adapted Scale

Completing the SEIQoL-DW can take up to one hour which may confuse or tire some frail participants. Moreover, successful completion of SEIQoL-DW requires reasonable eyesight and a certain degree of manual dexterity, lacking in some older people (24). In order to prevent this problem a simplified version of the instrument was developed that could be incorporated in an interview-administered questionnaire (see Appendix 1). The SEIQoL-Adapted provides an overall score for subjective quality of life, ranging from 25-100, with high scores indicating high quality of life.

### Procedure

ECH scheme managers were contacted for permission to use their scheme for fieldwork. Individual scheme managers who consented for their scheme to take part in the study were asked for permission to approach scheme residents as potential participants. The face-to-face questionnaire-based interviews took approximately one hour to complete on average and were conducted between March 2009 and December 2009 in the participants' living units.

### Data analysis

Before commencing quantitative methods of data analysis by using Statistical Package for the Social Sciences (SPSS v.16), three aspects of data including cases, variables and values were defined. To find a relationship between quality of life and use of technology, responses to the use of devices items were combined to create two main values, 'use' and 'non-use'. Then the use of HBT devices was categorised into 'low use' (participants who used less than six multiple household devices) and 'high use' (respondents who used more than six devices). Histograms and frequency distributions of the variables were examined to evaluate the normality of their distributions. The score ranges and distributions for all of the measures were examined using scatter plots. Chi-square test was used to investigate the possible relationships between technology use and gender, marital and health status. The independent samples *t*-test was employed to compare the values of the means of CASP-19 and SEIQoL-adapted scores amongst low users and high users of HBT devices. Regression analysis was used to explore the correlation between number of devices used and SEIQoL-Adapted and CASP-19 scores. Furthermore, multiple regression analysis adjusted for confounders including age, sex, marital status, living arrangement and mobility use was applied. For all tests, the alpha level for statistical significance was *p *<. 0.05.

### Ethical considerations

Ethical approval was sought and obtained from the University of Sheffield, School of Architecture Ethical Review Committee.

## Results

### Socio-demographic characteristics

Broadly speaking, most participants were female, aged 75 and upwards with good or excellent health, living alone, and white British. Most of the residents also lived in a suburban/urban area, small size schemes, and new buildings (see Table 1).

**Table 1:** Socio-demographic characteristics of participants (n = 160)

Characteristic/Frequency and percentage					
**Gender**					

Women 105 (66%)		Men 55 (34%)			

**Age group**					

55-64 14 (9%)	65-74 31 (19%)	75-84 69 (43%)	85 and over 43 (27%)	Not known 3 (2%)	

**Marital status**					

Widowed 87 (54%)	Married or cohabited 41 (26%)	Divorced or separated 17 (11%)		Single (never married) 15 (9%)	

**Ethnicity**					

White (UK or other) 159 (99%)		Asian or Asian British 1(1%)			

**Living arrangement**					

Alone 118 (74%)	With spouse 35 (22%)	With another person 5 (3%)		With spouse and other person 2 (1%)	

**Overall health condition**					

Excellent 8 (5%)	Very good 27 (17%)	Good 64 (40%)	Poor 56 (35%)	Very poor 3 (2%)	Not known 2 (1%)

**Mobility use**					

Wheelchair 18 (11%)	Wheelchair and other mobility aids 48 (30%)		Other mobility aids 55 (34%)	No mobility aids 39 (25%)	

**Scheme type**					

New build 124 (78%)			Remodeled 36 (22%)		

**Scheme location**					

Suburban 82 (51%)		Rural 58 (36%)		Urban 20 (13%)	

**Scheme size**					

Small 126 (79%)			Village 34 (21%)		

### Use of Devices

Basic appliances were used widely as everyday technology. As telehealth facilities, automatic temperature thermostats, property exit sensors, electronic window openers were on the whole not supplied by the schemes, very few respondents used these devices on any occasion. Among the respondents, 103 (64%) did not use washing machines in the scheme's laundry rooms, one-half did not use the assisted-bathing facilities, and around one-third of participants did not use the personal computers in their schemes, electric ovens, electric hobs and emergency call systems. Only 13 per cent of respondents (n = 20) used personal computers in their schemes. The results are summarised in Table 2.

**Table 2:** Use of HBT devices (n = 160)

Device	Frequency of Use							
	
	Everyday	2-3 times a week	Once a week	Once a month	<once a month	Never	Not applicable	Not Known
Electric kettles	140(87%)	2(1%)	1(1%)	0	1(1%)	12(7%)	3(2%)	1(1%)

Lifts	95(60%)	15(9%)	15(9%)	6(4%)	5(3%)	22(14%)	0	2(1%)

Microwaves	66(41%)	34(21%)	7(4%)	3(2%)	6(4%)	24(15%)	19(12%)	1(1%)

Toasters	55(35%)	52(32%)	8(5%)	5(3%)	7(4%)	22(14%)	10(6%)	1(1%)

Electric hobs	33(21%)	31(19%)	17(10%)	10(6%)	11(7%)	38(24%)	19(12%)	1(1%)

Electric ovens	21(13%)	32(20%)	12(7%)	6(4%)	20(12%)	46(29%)	22(14%)	1(1%)

Personal computers (flat)	21(13%)	8(5%)	1(1%)	0	5(3%)	5(3%)	119(74%)	1(1%)

Emergency call systems	10 (6%)	6(4%)	7(4%)	12(8%)	69(43%)	52(33%)	2(1%)	2(1%)

Washing machines (laundry)	7(4%)	14(9%)	13(8%)	11%)	3(2%)	103(64%)	18(11%)	1(1%)

Washing machines (flat)	5(3%)	21(13%)	9(6%)	3(2%)	5(3%)	14 (9%)	102(63%)	1(1%)

Closed- Circuit Televisions	4 (2%)	3 (2%)	3 (2%)	2 (1%)	8 (5%)	48(31%)	90(56%)	2(1%)

Personal computers (scheme)	3(2%)	1(1%)	3(2%)	0	13(8%)	58(36%)	80(50%)	2(1%)

Electronic window openers	1(1%)	1(1%)	0	1(1%)	1(1%)	5 (3%)	149 (93%)	2(1%)

Assisted- bathing facilities	1(1%)	7(4%)	10(6%)	1(1%)	19(12%)	82(51%)	38(24%)	2(1%)

Property exit sensors	0	0	0	0	0	0	158(99%)	2(1%)

Automatic temperature thermostats	0	0	0	0	0	4(2%)	152(96%)	4(2%)

Telehealth facilities	0	0	0	0	0	3(2%)	155(97%)	2(1%)

### Bivariate associations

Chi-squared tests revealed that there were no significant relationships between low/high users of multiple HBT devices and health status (*p *= 0.25), technology use and gender (*p *= 0.37) and technology use and marital status (*p *= 0.06).

### Use of technology devices and quality of life

For the SEIQoL-Adapted, M = 86.6, SD = 13.9, range 34-100. A linear regression applying technology use to predict SEIQoL scores showed a highly significant relationship. The coefficient was 1.29 with 95% CI (0.24, 2.34) and *p *= 0.016. This means that an increase of one device is associated with an increase of 1.29 points in total quality of life scores. Furthermore, multiple regression analysis adjusted for confounders including age, sex, marital status, living arrangement and mobility use indicated a coefficient of 1.17 with 95% CI (0.05, 2.29) and *p *= 0.04.

For the CASP-19, M = 39.3, SD = 10.1, range 14-56. A similar analysis, linear regression, for the CASP-19 confirmed a coefficient of 1.27 (0.50, 2.04) and *p *= 0.001. Interestingly, multiple regression analysis adjusted for confounders including age, sex, marital status, living arrangement and mobility use resulted a coefficient of 2.83 with 95% CI (1.17, 4.50) and *p *= 0.001. These results reveal that there was a positive association of the use of HBT devices to quality of life amongst participants living in extra care housing schemes in England.

Moreover, the independent samples *t*-test between CASP-19 and technology use showed that CASP scores were significantly different between high and low technology users (t = 3.32, df = 153, *p *= 0.001; low users *M *= 36.2 and high users *M *= 41.6). The same test with the SEIQoL also showed the same result as t = 2.2, df = 156, *p *= 0.02; low users *M *= 84.04 and high users *M *= 88.91).

**Figure 1:**
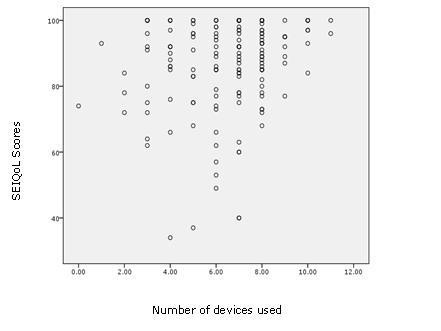
**Scatter plot of SEIQoL scores and the number of devices used**.

**Figure 2:**
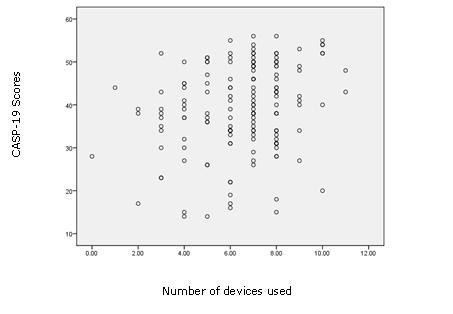
**Scatter plot of CASP-19 scores and the number of devices used**.

Finally, a simple scatter plot of SEIQol/CASP-19 scores against technology use (see Figures 1 and 2) showed a relationship, with quality of life generally higher among participants who used more devices.

## Discussion

In this study, we investigated the use of HBT devices and the correlation between use of such devices and quality of life among older people living in ECH schemes. With regard to the participants' socio-demographic characteristics, they were mostly white British (99%), aged 75 and over (70%), widowed (54%), living alone (74%), and living in urban/suburban areas (64%) in new purpose-built schemes. Seventy-five per cent of the participants used wheelchairs or other mobility aids, with a range of good-excellent self-reported health conditions (62%).

Our findings showed that none of the participants used exit sensors, automatic temperature thermostats, and telehealth facilities. In spite of the fact that many schemes were equipped with HBT devices, 73% of participants who had access to washing machines did not use them, followed by assisted-bathing facilities (68%), emergency call systems and ovens (33%), and electric hobs (27%).

The usage of IT and electronic devices was studied by Tomita and colleagues in 2004 [28] among 1,121 community-based Japanese older people. The results revealed that 89 per cent used microwave ovens, followed by rice cookers (83%), cash machines (83%), cellular phones (37%), personal computers (17%), and the Internet (12%). In comparison, the results of the present study indicated that the use of microwaves was lower at 72 per cent. In contrast, the use of personal computers was higher than in the Japanese sample. Twenty-five percent of the extra-care housing residents used personal computers connected to the Internet and in their living units.

The results of our study concluded that the use of multiple HBT devices is associated with better quality of life of among the residents of ECH schemes. Although the evidence base for the impact of basic technology is limited, previous studies have focused mostly on the relationship between age-related changes in visual, perceptual, motor and cognitive abilities and older adults' use and adaptability to new and assistive devices for disabled seniors or those with a medical condition [29]. Others have examined the efficacy of specific devices in enhancing functioning, reducing isolation, and improving quality of life [[30,31] and [32]]. Additionally, a recent line of inquiry has considered the extent to which assistive technology substitutes or supplements for personal care [[13] and 33]. By contrast, a great deal has been published about the role of computer-based technologies in influencing social aspects of quality of life.

Data analysis showed that health status, gender, and marital status were not significantly associated with the use of multiple HBT devices. Similar results were reported by Hartke and colleagues in 1998 [34]. Tomita and colleagues also stated that there is no general agreement about the findings of the relationships between gender, age, income, health status and the use of assistive devices. *"This may be due in part to the types of device in the studies, the definition of frail elders, the sample size, the region, and the types of categorisation in variables" *[[28], p.143].

## Strengths and limitations of the study

This is the first study in the UK that has been carried out to evaluate the use of home-based technology devices in occupied ECH schemes.

Some difficulties arose in data collection. For example, words may have different meanings for different age groups particularly. The use of technical terms such as technology', 'telehealth', and 'sensor' was difficult to avoid, and some terms were difficult to describe.

As the data collected in this study is from a specific, new form of housing for older people, the results of this study cannot be generalised to all elderly people living in other forms of housing, particularly those suffering from cognitive impairments. Additionally, few participants used or had access to some of assistive devices making analysis of their characteristics impossible. Furthermore, the required sample size was not obtained, meaning that the analyses reported are slightly under-powered. There is therefore an increased risk of Type II errors.

Although the correlation between use of HBT devices and quality of life was mostly based on multiple regression analysis adjusted for confounders including age, sex, marital status, living arrangement and mobility use, there are several variables such as depression, comorbidity and frailty- not considered in this study- which could be mediators or confounders.

## Conclusions

The findings of the present study will be of value to those who are developing new forms of specialised housing for older people with functional limitations and, in particular, guiding investments in technological aids. The results of the present study also indicate that the home is an essential site for developing residential technologies.

As older people's interest in new technologies grows, further studies on technology acceptance amongst elderly people will be usefully informed by the findings of this study. Future multidisciplinary research may focus more deeply than the present study on the barriers influencing the use (and non-use) of technology.

## Competing interests

The authors declare that they have no competing interests.

## Authors' contributions

HM developed an original complementary study specifically for his doctoral thesis and carried it out with the guidance of KM, SGP, Chris Parker, and contribution of 'evaluation of older people's living environments' (EVOLVE) project. All authors read and approved the final manuscript

## Appendix 1: Procedures for use of the SEIQoL-Adapted

### First guideline: using the scale

1. Name five items that are very important for your quality of life;

2. Which of these items are the most important for you that would you put in first place and then second place and so on?

3. How satisfied you are with each item? You can be 'very satisfied', 'satisfied', 'dissatisfied', or 'very dissatisfied'.

### Second guideline: scoring the scale

1. The responses to question three are then ranked as very satisfied = 4, satisfied = 3, dissatisfied = 2, and very dissatisfied = 1.

2. Some respondents provided answers on one item, some on two, some on three, four and five. The distribution of the overall quality of life scores for those providing answers to different number of items were all standardised to a range from 25 to 100, as follows:

If the participants named one thing that was important for their quality of life then the overall score (S) was:

S = 25 × (A) where A is the rank (between 1 and 4) of item;

If the participants named two items then the overall score was:

S = 15 × (A) + 10 × (B) where A and B are the ranks of the first and second items;

If the participants named three items then the overall score was:

S = 12 × (A) + 8 × (B) + 5 × (C) where A, B and C are the ranks of first three items;

If the participants named four items then the overall score was:

S = 10 × (A) + 7 × (B) + 5 × (C) + 3 × (D) where A, B, C and D are the ranks of the first four items;

If the participants named five things that were important for their quality of life then the overall score was:

S = 10 × (A) + 7 × (B) + 5 × (C) + 2 × (D) + 1 × (E) where A, B, C, D and E are the ranks of the five items.

For example, if a participant answered three items and were ranked 2 (dissatisfied),

3 (satisfied) and 1 (very dissatisfied) the overall quality of life score would be S = 12 (2) + 8 (3) + 5 (1) = 53.

## Pre-publication history

The pre-publication history for this paper can be accessed here:

http://www.biomedcentral.com/1471-2318/11/68/prepub
